# Things matter: about materiality and recovery from mental health difficulties

**DOI:** 10.1080/17482631.2020.1802909

**Published:** 2020-08-03

**Authors:** Inger Beate Larsen, Tore Dag Bøe, Alain Topor

**Affiliations:** Department of Psychosocial Health, University of Agder, Grimstad, Norway

**Keywords:** Recovery, human becoming, material turning point, meshwork, mental health

## Abstract

**Purpose:**

The aim of this study is to explore how material things might become involved in the recovery process of people with mental health difficulties.

**Method:**

Empirical material from three different studies on various aspects concerning mental health issues that each of the authors had conducted was reanalysed through a phenomenological item analysis.

**Results:**

We discovered that mundane objects such as a mobile phone, a bench, a door and a key have agency to contribute to peoples’ recovery and wellbeing. Things became agents that created contexts that initiated physical, social and emotional movements.

**Conclusion:**

By giving attention to materiality we might become aware of the importance of things as agents in living in general and in recovery processes for people with mental health difficulties in particular.

## Introduction

This study concerns material things that may be important for the recovery process among people with mental health difficulties. Our intention is to study how material objects influence and participate in this process. More specifically, we investigate how people use objects for physical, social, and emotional movements in their life and mental wellbeing, but also how things themselves have agency to make differences in people’s life.

Gumbrecht (2004) points out how human sciences seem to be bound up in hermeneutic perspectives; that is, an interest in the way humans understand and make meaning. Thus, Gumbrecht (2004) implies that the materiality of human lives, both the material surroundings and the materiality of the living body itself, is forgotten. He suggests that this hermeneutic world view in fact entails a “loss of the world” (p. 49), which ignores materiality. Hammersley and Atkinson (2007) emphasize how social research tends to ignore the role of material artefacts: “[…] everyday life demands attention to its material features, and how social actors engage with physical things” (p. 134). “Social actors” here refers to humans, but we believe physical things are intertwined in social life in such ways that the things themselves could also be described as social actors.^1^

Ingold (2010, 2013a, 2013b) replaces traditional models of genetic and cultural transmission with environmental perception and skilled practices that occur in relation to one another. This relational approach focuses on the ongoing growth of embodied skills of perception and action within social and environmental contexts of human development. Hence the knowledge and skills of humans are in fundamental ways embedded in, and emerge from, interplay with material environments and cannot be thought of in terms of transmission of ready-made knowledge or skills from one generation to another. In fact, Ingold (2013b) suggests a new paradigm in human sciences where the concept of “human *beings*” is replaced by the concept of “human *becomings*”. Ingold writes that such a paradigm of becoming (as opposed to being) introduces an:

[…] entirely different ontological foundation. We can no longer think of the human as a discrete, bounded entity, set over against the environment. It is rather a locus of growth within a field of relations traced out in *flows of materials*. (Ingold, 2013b, p. 10)

Research on materiality connected to place, space and things, shows e.g. that a Lego brick and a medication box enter into relationship with the people who have everyday contact with the items, and become part of their construction of identities, of the professional languages and also of the structure of space and timing (Larsen & Melhuus, 2016). Goffman (1968) shows that materiality influences the practical life in total institutions. In particular, social and health geography have contributed to putting the power of place, space, and things in focus (Larsen & Topor, 2017; Bøe et al., 2019; Holloway & Valentine, 2000; Parr, 2006, 2008). Newer materiality theory can be understood as a critique of the constructivist and discourse analytical one-sided focus on language, and also as demonstrating a shift from what materiality does to us to “how materiality is done” as Damsholt et al. (2009) articulate it. Studying “how materiality is done” connects objects to history and culture and recognizes that things make us as much as we make things. Materiality and people “are done” in various ways in and through *correspondence*, to use a term from Ingold (2017).

The present study focuses on important things for people with mental health difficulties and discusses how materiality is done using the descriptions about meaningful objects from the perspective of the people themselves. Hanghøj (2005), Mordhorst (2009), and Otto (2005) each have a research perspective which focuses on specific selected items, like Otto (2005) research on how an old lady’s sofa pillows were connected to identity and memories about younger days. Bennett (2010) shows how things relate to the environment in powerful, material assemblages, meaning that the relationship between humans and materiality should be read as a blending rather than vertically as a hierarchy of being.

In addition, we use the concept of recovery, which refers to a process of regaining control over one’s own life, through one’s own efforts and support from both one’s formal and informal social network (Topor et al., 2011). Recovery is also about regaining meaning in everyday life (Borg, 2007; Borg et al., 2005), and is described as both an individual (Anthony, 1993; Deegan, 1996) and a social process (Mezzina et al., 2006; Tew et al., 2012). A social process was necessary to become citizens in people’s own communities, rather than being “people with mental health problems” (Mezzina et al., 2006). However, the significance of materiality is seldom described in recovery literature, although Borg et al. (2005) found that “in several instances, informants’ references to material resources were implicit in their discussions of activities that fostered recovery” (p. 245) something which is important in our study because we want to make materiality explicit. We therefore pose the following research question:

In which ways might material things become involved in the recovery process of people with mental health difficulties?

## Research design

The authors originally studied various aspects concerning mental health issues. Topor studied recovery processes in a project including interviews with 30 people earlier diagnosed with severe mental illness. At the time of the interviews, they defined themselves as in recovery or fully recovered and had not been admitted to a psychiatric institution for at least the last two years (Schön et al., 2009; Topor, 2001; Topor & Denhov, 2015). Larsen studied the impact that surroundings, buildings, rooms and objects in Norwegian institutions had on patients and staff. She conducted participant observation in five district psychiatric centres and additionally interviewed 16 patients and 22 professionals (Larsen, 2009). Bøe studied processes of change among Norwegian adolescents (16–18 years old) who received mental health care from a hospital out-patient service. This study was based on qualitative interviews with eight adolescents, eight persons from their family and social network and six practitioners^2^ (Bøe et al., 2013, 2014, 2015). Thus, all the authors had data based on the perspective of people with mental health difficulties. In these separate research projects, we became aware of findings connected to the significance of small things. We decided to do a second analysis of some of the data to acquire knowledge of how *small things* as gestures, words and physical objects might be of importance for peoples’ wellbeing (Bøe et al., 2019), because we perceived that this perspective was not given the proper attention so far (Topor et al., 2018).

In this present study we give a more elaborate and nuanced understanding of the roles of material things and focus on the interaction between people and material things.

### Data

The data consist of transcribed interviews from the three different research projects. We chose to reanalyse the text from four interviews from each project. Since Larsen had studied the impact materiality (like surroundings, buildings, rooms and objects) had on patients (and staff) in Community-Based Mental Health Centres (CBMHC), almost all her data gave a first-person perspective on how materiality impacted patients’ lives. As Bøe and Topor did not explicitly focus on material things in their studies we chose interviews that referenced material things. Thus, we selected 12 interviews, eight with adults and four with adolescents. In addition, we included field notes from Larsen’s research since she also performed participant observations. As the field notes also consisted of data about the material surroundings, we chose sequences from this text that involved material things (Larsen, 2009). Altogether we had 162 pages of text from the interviews and field notes to re-analyse.

### Method

A phenomenological item analysis^3^ combined with constructionism was used (Hanghøj, 2005). According to Hanghøj the main intention of this method is to understand objects as parts of contexts and as objects which create contexts, meaning understanding objects as actors. Thus, we were looking for descriptions of how objects were used, but also of how the objects themselves made people act. Therefore, data about the relations between people with mental health difficulties and objects are highlighted both in the interviews and in the field notes. By involving materiality, we turned away from representations that focus on experiences. Instead we looked for material realities and how people with mental health difficulties cope with the world (Otto, 2005). Inspired by Damsholt et al. (2009) and Ingold and Palsson (2013) we stressed a focus on *becoming* rather than *being*, by focusing on the objects as agents and the way they influenced the participants in their “ongoing becoming.”

The material turn implies a method concentrating on items and people and asking questions about “what the material does in the world and how the material is done in concrete time and spatial contexts” (Damsholt et al., 2009, p. 13) (authors’ translation).

### Analysis

All three authors read through the shared data and had preliminary discussions. We then re-read the texts both from the interviews and field notes and identified “key moments” (Sullivan, 2012), where material things were mentioned. A key moment, as suggested by Sullivan, is a sequence of utterances in which there is a significant meaning unit. An illustration of a key moment shows how a door became involved in a young boy’s story of going back to school: “To walk through that door, it was like …, It was like going to my mother’s funeral. It was completely crazy”.

In our case, we identified things that seemed important according to those interviewed. Field notes supplied more information about the context and atmosphere and of how the participants and objects interacted.

Altogether we found 13 things which could be defined as parts of “key moments”: living room, flowers, plants, tablecloth, sofa, chair, uniform, room, bed, mobile phone, bench, door and key. We then met and discussed which of these objects we should investigate more closely. Based on our item analysis (Hanghøj, 2005), we chose items which were both parts of contexts and which created contexts. We chose to emphasize the following items: mobile phone, bench, door, and key. The reason for this decision was that we had contextual data about the interaction between the participants and these material objects. And also, because these objects were part of the participants’ stories about wellbeing, challenges, and recovery. In the discussion we made a thorough investigation of each selected object. In the beginning we noticed that we had difficulties with the material turn, and we often had to correct ourselves because we forgot the focus on becoming, and were more interested in how the participants used the objects than how the objects became actors in their lives.

We decided to describe the four objects as concrete, physical phenomena. By doing so we became aware that such descriptions helped us to better understand the items as agents and how materiality was done. For example, a mobile phone was not only an object, but every application was a connection between an owner and other people or other functions in different contexts. A key was not only a thing, but an actor with possibilities for control.

Further on we re-read the text about the interaction between people, the chosen objects, and the meaning of recovery (Larsen & Hohl, 2015; Brinkmann & Kvale, 2015). We then aimed at detecting patterns, themes, repetitions, contrasts, and paradoxes in the material (Brinkmann & Kvale, 2015) in order to grasp the meaning of these interactions. Consequently, the analyses could give a more informed view of recovery processes, and whether these processes and the agency of the chosen items could be related to recovery. The analysis process also emphasized the interactions between people and objects and the kinds of functions these interactions might have for the people to whom the objects had significance.

### Ethical considerations

All three studies that provided data for this present study were conducted according to legal and ethical principles for research. Bøe and colleagues’ and Larsen’s studies were approved by the Norwegian Regional Committees for Medical and Health Research Ethics (ref. S-03073 and 2973–2). Larsen’s study was also approved by the Norwegian Centre for Research Data (ref. 9925). Topor and colleagues’ study was approved by the Department of Social Work at the University of Stockholm. Participants in all studies gave their informed written consent to participate. In Bøe and colleagues’ study, the participants were 16–18 years old and their parents/guardians also gave their written consent. The interviews shared between the authors in this present study were all anonymized.

### Strengths and limitations

We are fully aware that the reanalysis of the interviews from the studies that did not explicitly focus on material things may have a flaw because two of us did not ask people directly about the objects. On the other hand, the data show that objects are important because they are mentioned several times without being asked about. We are also aware that most objects are collections of other things (Bennett, 2010) and hence are hard to interpret as separate objects. Nevertheless, we think that focusing explicitly on specific objects may be an important supplement to the existing recovery literature.

The fact that we are three different researchers with different backgrounds has of course also influenced the analysis. Topor is a psychologist; Bøe is a social worker and Larsen is a registered nurse. We may interpret these different professional backgrounds as a supplement to each other that has made us able to perform a more nuanced and informed analysis than we could have done alone. We also discussed why two of the authors did not focus on materiality in their original projects, and this is difficult to understand when we have now noticed the clear presence of objects. However, the studies of Bøe and Topor both explored the recovery processes from the experiential perspective of the participants. These studies did not have a pre-chosen focus on materiality as the aim was to be open to all aspects that seemed significant to the participants in their lives.

## Findings

We will now present data about how a mobile phone, a bench, a door, and a key may be involved in the lives and recovery processes of people with mental health difficulties.

We start by giving concrete descriptions of each item. These descriptions are our own subjective presentations, and the picture we give should be considered as general descriptions of the chosen objects. Secondly, we present descriptions and key moments according to the participants in the three different projects.

### Mobile phone

The mobile phone we are talking about is a smart phone. It is a portable telephone, usually made of plastic and metal in assorted colours. The owner can make and receive calls while he/she is moving or sitting still. In addition, the mobile phone provides other services, such as text messaging, MMS, email, internet access, video games, and photography. Being in possession of a mobile phone, people are able to listen to the radio, watch television and listen to music. A mobile phone is quite small, and you can keep it in your pocket. The mobile phone might be a status symbol. Brands, colours, shapes, and covers communicate identity and connectedness to others. In that way a mobile phone is a material thing with a lot of possibilities of influencing people’s ongoing becoming in various ways.

Our data shows that owning a smart phone may reduce feelings of displacement, loneliness, and anxiety. Having one meant that, in some way, the person was connected to a better, less anxious place. A young girl experienced her mobile phone as an object that made her less lonely, because it connected her to other people that meant a lot to her: “I always had somebody; somebody I could call. I have always had some […] I have in a way always had somebody there for me” (B^4^).

A boy had the same kind of experience. He said that his phone was something that helped him in demanding situations. For him it was emotionally very hard to go to school, he was always afraid of making a fool of himself and he felt like an outsider in the classroom. But he knew he had to go to school, and the mobile phone made it easier when he felt lost: “After a while I could barely get by. I then called my dad and passed the phone over to the teacher, so he could be aware of it. And then […] after all I had someone to talk to” (B).

Since the mobile phone was also an instrument that made it possible to listen to music, the phone helped him to cope with a stressful situation:

I still remember which song I was listening to […]. I was scared to death […]. Yes, it was when I was sitting on the bus and […]. The song I was listening to, it was, very […] it was two songs. […] No, in fact three. Three very good songs. Like, they have meant a lot to me throughout time. One has been in ‘Paradise Hotel’ (B).

An extension of the phone is the headset, and this boy continued:

I brought my headset [on the bus]. I have Spotify […]. Hmm, but I just thought a little taste (in the interview he starts playing from his mobile). This is a competition song […] in fact a song for celebrations […]. I knew it would work out well because I had that song on when I walked in that door (B).

He was afraid of going to school and on his way the mobile phone with the headset offered him music that helped him when meeting other pupils, even if the meetings were challenging for him. One young female patient in Larsen’s study at the CBMHC also listened to music in challenging situations. She told that when she felt bad, she always picked up her phone and turned on some music. For her the mobile phone became a kind of actor that “[…] helps for restlessness and anxiety. It’s a kind of relaxing music. At the same time, you get time on your own. That helped a lot. For the anxiety. So that’s great.” (L), she said.

For a young male patient in another CBMHC the mobile phone also helped him to relax, even if it came at a cost for him. He told the following:

I am addicted to games. To play data games. It’s like the staff think they have nothing to do with it, but personally I think it should be forbidden. But it is like […]. I can get addicted to a lot of odd stuff now, so it is better to be allowed to play to calm down. Otherwise I go crazy (L).

Larsen observed that this patient was often sitting alone in the corridor holding his mobile phone in his hand all the time. He seemed eager when he played a game, but it was not difficult to ask him for a chat. When talking to someone, he put his phone in his pocket. In this way the games could be a replacement for relationships with other people, or it could be an excuse to avoid others.

### Bench

A bench has a structure of wood, stone, metal, or plastic. Bench colours vary. A bench has a long seat on which several people can sit side by side, outdoors or inside a house. People can sit in silence; they can rest; they can wait for something to happen or for somebody; or they can talk to others sitting on the same bench. A bench may be a symbol of attention and may encourage people to talk. A bench can also offer the possibility for people to lie down and rest or sleep. We do not always know exactly the kind of bench to which the different participants referred (wood, concrete, metal), but we do know they were all outdoor benches, and of course the material they were made of would matter for people’s wellbeing. Wood is softer than concrete. Metal is colder than wood.

In our data we found that benches connected people to other people something which may be seen as improving wellbeing. A young girl in Bøe and colleagues study, had for a long time felt that she did not fit in at school. She said she was bullied, and she always sat alone in breaks. She started at a new school and experienced being included there. A bench became an important part of her inclusion process:

And I have in a way gotten much better contact with everybody, and the atmosphere in the classroom is nice. I feel I know almost everyone so if I go out and somebody is sitting on the bench I can go there and talk to them and usually they know who I am. (B).

This bench was placed in the school yard. As an agent, the bench itself encouraged the pupils to sit down, to be close physically and mentally. The bench communicated togetherness and inclusion. On the other hand, the bench could of course also represent estrangement, and people could feel excluded if nobody invited them to sit down.

In Larsen’s (2009) research a specific wooden bench was observed as a key place outside of a CBMHC building. She noticed four patients sitting close to each other on that bench. Earlier that day she had heard a discussion among the staff about patients diagnosed with psychosis. They agreed that the patients were persons who were not able to be physically close to other people. The staff referred to well-known psychoanalytic literature. Therefore, Larsen went to the patients on the bench, referring to the literature and said. “I have read that people with problems like yours prefer not to be physically close to other people, so how come you are sitting that close?” All of them laughed and one replied “Don’t they think we are human beings? All people need to be close to others.” In a way, the bench confirmed the patients as persons with the same needs as everybody else instead of somebody who are too vulnerable to be closely related to others.

### Door

A door covers an opening in a wall or in a piece of furniture and is usually made of wood and sometimes of metal. It is a moving mechanism used to block off and allow or hinder access to entrances to or within an enclosed space, such as a building, a room, or a cupboard. Doors normally consist of one or two solid panels, with or without windows, that swing on hinges horizontally. Sometimes you need a key to open a door. The main purpose of a door is to control physical access. If you open a door, you may, or may not, know what is behind it. A door can be an exit to see something familiar or something new. An open front door can let in the light, and a closed door may make a room dark.

In our data we found that doors represented all the aforementioned elements: access, concealment, the unknown. Returning to the boy (presented above) who was helped by his mobile phone, it seemed that the front door into his school represented a great difficulty to him. It was literally the entrance to an unknown, unpredictable and frightening world where he felt he had no longer control. “But it was … To walk through that door, at that time I hadn’t talked with anybody. I didn’t know the teacher, I knew nothing. To walk through that door, it was like …, It was like going to my mother’s funeral. It was completely crazy” (B). To walk through the door was to him like doing something he had never done before. He would meet people he did not know; he did not know how they would act towards him, or how he would react. Behind that door he felt he had absolutely no control.

In different CBMHC the doors into the staff offices became important. Larsen observed that many patients were sitting still or wandering around waiting for the staff to enter the door. Many patients talked about these doors as “doors to the unknown”. A male patient said the following about the closed door to the staff office:

It’s a clear lockout. Because there are no windows […] it’s a completely closed room. And we are scarcely allowed to put a foot over the doorstep when we need medication and stuff. So, myself I feel it’s a big rejection. I’ve tried to not think of what they are saying about me inside there. […] but that room, and that door, may be a great snag. […]. It’s very frustrating. [It would be nice] to open [the door to the] charge room a little bit, so it was not sort of so secret.” (L).

At one CBMHC office, notes on the doors underpinned the secrecy. On one door it said: “Only for staff”. On another door a note said: “Be so kind as to respect the timetable for reports”. The staff said that they used the closed door as a therapeutic instrument to help the patients back to society. They said that “out in the world” one has to knock at the door of the social services, of the medical practitioner or of the employment office. It is noteworthy that the staff used doors to represent “real life” and consequently the staff room door represented all kinds of official doors.

The restless atmosphere that Larsen felt when the door was closed disappeared when the staff came out from the office door. The patients seemed to relax when they could be together with the staff again, as the open door made it possible for the two groups to meet.

### Key

A key is usually made of metal and makes it possible to turn the bolt of a lock. This small item is a tool for opening and locking doors. People with their own homes, cars, or cabinets, also have their own keys to get access to these things. These keys are to be understood as symbols of identity and give control of one’s personal life.

In our data, we found that keys represented identities of normality, inclusion, and control. For several participants, a key was an important object. A man who had earlier been a patient for years, remembered well the day he got a key to the ward where he was still a patient, as he also started working as an assistant at the same hospital: “I went down to […] and had my own key, of course no medicine cabinet key but I had a key to the doors and thus I had an identity” (T). Here, the agency of the key was to give him a new identity. Of course, it was not the key alone that did this, but the possession of the key while he still was a patient changed him. He was not just a patient anymore, but a patient with the key to the ward: a paradoxical identity of patient and staff member at the same time.

A young lady from a CBMHC mentioned the possibilities to lock and unlock doors, without being dependent of the staff:

Yeah, we have keys to the room, and we [all the patients] also have keys to the wardrobe in our rooms. And to a small security box inside the wardrobe […] for articles of value and that kind of stuff. That is quite OK. But of course, the staff have keys in case something should happen (L).

In this case the keys gave her access to her room and made it possible for her to lock the door when she was inside or when she went out. The key gave her control over her personal belongings. But since the staff also had keys, these keys had more power than those of the patients: “If something happened” the staff could unlock their doors with their universal key and walk into the patients’ [bed]rooms (L).

## Discussion

We have showed that objects can be seen as agents of physical, social, and emotional movements. In each instance we analysed, we found that people used objects to connect with other people, places, spaces, and things. And we mean that these objects in themselves also made people act in various ways.

Living inevitably implies moving around in a world of material things. We approach them, move away from them, touch them, use them, interact with them, interact with other humans through using them. Our intention is to discuss how material objects influence and participate in peoples’ lives and focus on how certain things may have agency and contribute to peoples’ recovery.

Our main finding was that material things have an impact on peoples’ lives and recovery processes. Things became agents that created contexts (Hanghøj, 2005); these contexts, constituted by things, allowed people to act and move in the ways they did. The objects we investigated illustrated how things entered into intertwined relationships with the participants and also defined them. The mobile phone, the bench, the door, and the key can be seen as actors that made it possible for people to move something which then formed and defined them. The different objects gave them the ability to lead lives as ordinary citizens something which is an important ingredient in social recovery as being an ordinary citizen is a goal to attain and quite different than being a patient who lives with mental health issues and needs medical interventions (Tew, 2013). This finding is particularly important as moving from being mentally ill to being a citizen like anybody else (Topor et al., 2011). Studies show that some things, e.g. a medication box, may affect persons in such ways that they identify themselves—and are identified by others—as sick persons (Larsen & Melhuus, 2016). At the same time other things might affect persons in such ways that they identify themselves as citizens, like the example with the key to a working place which could be understood as an object that gave the man with mental health problems an identity as an ordinary citizen.

We will now discuss material things as agents of movements in the lives of people in general and thus in the lives of people with mental health difficulties. Ingold says that living as and becoming a person implies being open to the world, and in order to be open to the world we must “surrender something of our agency” (Ingold, 2017, p. 16). Following this we could suggest that humans are moved by (the agency of) things. So, we suggest that things could be thought of as having agency because it may help to point out how things that might be considered as unimportant dead material, seem to be able to influence and matter in the lives of the participants.

### Agents of physical movements

By focusing on material things as agents, we focus on becoming rather than being (Damsholt et al., 2009; Ingold, 2013b). When the boy walked through the door—in spite of his difficulties—one might perhaps say that his becoming is happening as he moves through that door. When you open a door, the door reveals the new world which you may enter, like the young boy felt on his way into the school. This physical movement may be an important step (literally speaking) in his recovery process and a personal strategy for managing an ongoing distress experience (Tew, 2013). The music from the mobile phone accompanied, or even assisted, the movement of the boy through the school’s front door and the bench became a helper, allowing the patients to come closer to each other so they could “feel human” or become human together. The key, for example, literally gave the man in the hospital possibilities to move in other directions than before. The doors, the rooms and the key opened up the surroundings for his becoming; new possibilities of growth within a “new field of relations”, to use Ingold’s words (Ingold, 2010, p. 3). Furthermore, the staff trusted him by giving him a key to the ward. And that specific key helped him to become someone having a tool (key) to new sorts of movements, into new contexts, that were previously impossible. He was able to come and go as he liked. He could move around in, and away from the hospital, and he could return. Thus, he could reclaim a positive “place in the world” (Tew, 2013, p. 362). As such an object has agency to make people move, and as agents, keys and doors for example, invite to physical, bodily movements towards a new future that may still hang in the balance.

### Agents of social movements

Ingold (2017) proposes the concept of *correspondence* to describe the dynamics of human living. He suggests that a human being should not be thought of as an entity that relates to other entities (humans or things). If we think of human becoming as movement, then movements form a line or a bundle of lines. These lines of movement are formed in responsiveness to other lines of movement:

Limbs move, stones settle, timber binds, voices harmonize, and family members get along through the friction and tension in their affects. They are not ‘and … and … and’ but ‘with … with … with’. (…) In answering—or responding—to one another, they co-respond. Accordingly, I propose the term correspondence.. (p. 14)

As we can see, material things are equally parts of this correspondence (stones, timber) and this correspondence also constitutes social life. As he continues “Social life [is] correspondence (…) the process by which beings and *things* literally answer to one another over time.” (p. 14). Hence the bodily movement is also a social movement.

We can move things, and things allow us to move. The mobile phone was an object that allowed the participants to make movements in response to others. For one adolescent, his phone gave him access to a song that helped him into the classroom and into a social setting. It seems that without the song in his phone, he would not have been able to enter that room. The mobile phone allowed another boy to talk to his father from the school and the phone represented a kind of “relationship capital [that] involves having a significant other who can simply ‘be there’ for us consistently through our various ‘ups’ and ‘downs’” (Tew, 2013, p. 367).

The bench had potential to make people move and sit with others. Inclusion and togetherness happened in correspondence with a bench and some participants. “All people need to be close to others”, was a statement from a patient and illustrates the bench’s agency of making social movements. Davidson et al. (2001) talk about recovery as a help to increase the individual’s access to, and opportunities for relationships with others. Additionally, Tew (2013) highlights that crucial to recovery are social contexts that are non-stigmatizing and offer acceptance.

Our social identity is formed in response to objects. Many of these objects have been made through the social movement of making, and the made objects, in turn, affect our social movements and identity. The key, for example, may be a helper for a person with mental health difficulties to become a person rather than merely a patient. As one of the participants said: “[…] I had a key to the door and I thus had an identity”. When the man got the key, it made him feel like a citizen out in the world even though he was hospitalized. He gained some control over his life and rebuilt a positive identity, both of which are important recovery factors (Tew et al., 2012). Tew et al. (2012) conducted a review of research on recovery that clearly showed that social inputs may make a major difference in enabling recovery outcomes, particularly when there is a corresponding emphasis on empowerment, relationships, and social inclusion. All the items we investigated might be actors of social inclusion, as well as exclusion. In our data, the locked staff room door represented exclusion to the patients something which might have worked against their recovery processes, even though the staff saw the locked door as an invitation to gain experience in visiting public offices.

### Agents of emotional movements

Material objects give people possibilities to move, and in these movements, people will also move from one emotional state to another. In moving there is a continuous attention and responsiveness to the surroundings that we move into; the attention of seeing, listening, feeling. Becoming through movement is also a continuous emotional becoming (Bøe et al., 2014). We have seen that a movement from anxiety to comfort happened because of music from the phone: «That helped a lot. For the anxiety”, a participant said. We have also seen that a phone helped a lonely person to feel connected to other people and be less lonely. “I have in a way always had somebody there for me”, as another participant explained when talking about her mobile phone. Mobile phones have become important items in the world, and the way they have an impact on people is significant in concrete time and spatial contexts (Damsholt et al., 2009).

A door might also be connected to emotional movements. Walking through a specific door was not an easy feeling for the young boy on his way back to school; it felt like going to a funeral of somebody he loved. But this terrible feeling was reduced when he listened to music on the phone and made a phone call to his father. Mobile phones, benches, doors, and keys can be agents to help people become less restless, less lonely, calmer, and happier at the same time as they have the agency to do the opposite. As such agents they may also disturb in different ways, as the games on the phone did for the young boy who said he was addicted to games. On the one hand he wanted the staff to take away his phone, but on the other hand he became relaxed by using it.

## Conclusion

Our findings and discussion referred to specific material objects that fostered recovery and, we believe, made materiality explicit. At the same time, material objects have the ability to sometimes hinder movement and recovery.

In the discussion we have emphasized the agency of things that initiate physical, social, and emotional movements as if the different movements are separate from each other. But of course, this is a simplification. The different movements are an entanglement of “interwoven lines”, as Ingold (2013a, p. 132) puts it. And these lines “may loop or twist around one another or weave in and out.” Ingold suggests that every living being should be seen as a bundle of lines (Ingold, 2017, p. 10) and he writes that they do not necessarily connect as in a network. An alternative to a network is a “meshwork” of movement or growth:

“[The lines] are temporal ‘lines of becoming’. Life is a proliferation of loose ends. It can only be carried on in a world that is not fully joined up. Thus, the very continuity of life—its sustainability, in current jargon—depends on the fact that nothing ever quite fits.”. (Hallam & Ingold, 2014, p. 132)

And the things of the world and “the fluxes and flows of materials” (Ingold, 2010, p. 3) are fundamental in this meshwork of proliferated lines.

Living involves moving physically. Social life, we suggest, should also be understood in terms of physical and social movements, and furthermore the emotions that make life hurtful or joyful arise from these movements. The roles of things and the question of how things make us act perhaps deserve more attention in the research on recovery processes related to mental health. Since recovery is about gaining control and meaning, material things, which are meaningful to the person with mental health issues, will support physical, social, and emotional movements that support recovery.

The findings imply a widening of interest; questions of identity and roles are not the primary focus. Instead, questions on becoming and movement come to the fore. By giving attention to material things we may become aware of the importance of things as agents in living in general and in recovery processes for people with mental health difficulties in particular. If a bench is an agent of inclusion, keep it where it is placed. If it represents exclusion, then move it away. Or rather: Let the bench just be there, because it will certainly matter in different ways for different people.

## Notes

1. We will in the further presentation use actors and agents as synonymous.

2. For the purpose of this study only interviews with the adolescents were included.

3. Hanghøj writes in Danish and call this a “fænomenologisk genstandsanalyse”; a “phenomenological item analysis’ is our translation.

4. We mark the quotation with the first letter in the surnames of the authors (B, L & T) to show which of the researches material each extract comes from.
